# Infarctus médullaire aigu: un diagnostic méconnu au pronostic sévère

**DOI:** 10.11604/pamj.2018.31.85.16854

**Published:** 2018-10-04

**Authors:** Mohamed Amine Mnaili, Ahmed Bourazza

**Affiliations:** 1 Service de Neurologie, Hôpital Militaire d’Instruction Mohammed V, Rabat, Maroc

**Keywords:** Infarctus médullaire, syndrome médullaire aigu, embolies fibrocartilagineux, Medullary infarct, acute medullary syndrome, fibrocartilaginous embolism

## Abstract

Medullary infarct is still little known influencing the short-term vital and functional prognosis of patients. Medullary vascularization is one of the most complex in the body due to the significant number of arteries that contribute to it. Our knowledge is still limited due to the scarcity of vascular medullary accidents and to low accessibility of medullary vessels during assessments. We report the case of a 62-year old patient with type 2 diabetes and a 13-year history of oral antidiabetic treatment who had been followed-up for psoriasis for 4 years. He presented to hospital in the night with his son due to acute onset of upper limb deficit. This deficit was preceded by acute onset of neck pain during his daily prayer. On admission, the patient was conscious, arterial pressure 120/86mmhg, heart rate 89 beat/min, afebrile. Clinical examination showed flaccid brachial diplegia. There was no sensory disorder or sphincteric disorder and cranial nerve examination was normal. Emergency medullary MRI was performed which showed T2 hyperintense signal and “snake-eyes”-like bilateral central dissemination (A and B). Etiological assessment showed therosclerotic patches at the origin of the left internal carotid artery. The diagnosis of myocardial infarct associated with medullary fibrocartilaginous embolism was retained. Functional rehabilitation was started early as well as treatment with platelet aggregation inhibiting drugs.

## Image en médecine

L’infarctus médullaire est un diagnostic relativement méconnu, qui engage à court terme le pronostic, aussi bien vital que fonctionnel du patient. La vascularisation médullaire est l’une des plus complexes de l’organisme en raison du nombre important d’artères qui y contribuent. La rareté des accidents vasculaires médullaires et la faible accessibilité des vaisseaux médullaires aux investigations expliquent que nos connaissances restent limitées. Nous rapportons le cas d’un patient de 62 ans, diabétique de type 2 sous antidiabétiques oraux depuis 13 ans et suivi pour un psoriasis depuis 4 ans, a été amené à l’hôpital dans la nuit par son fils devant l’installation brutal d’un déficit des 2 membres supérieurs. Ce déficit était précédé de cervicalgie aiguë lors d’une montée brutale du patient en pratiquant sa prière quotidienne. A son admission, le patient était conscient, tension artérielle: 120/86mmhg, fréquence cardiaque: 89 battements/min, apyrétique. L’examen clinique révélait une diplégie brachiale flasque. Il n’y avait pas de troubles sensitifs ni de troubles sphinctériens et l’examen des paires crâniens était normal. Une IRM (Imagerie par Résonnance Magnétique) médullaire était réalisée en urgence, mettant en évidence un hyper signal T2 et une diffusion centrale bilatérale réalisant un aspect de « Snake-eyes » (A et B). Le bilan étiologique avait révélé la présence d’une plaque d’athérome à l’origine de l’artère carotide interne gauche. Le diagnostic d’infarctus médullaire sur embolies fibrocartilagineuses a été retenu. Une rééducation fonctionnelle est commencée précocement ainsi qu’un traitement par antiagrégant plaquettaire.

**Figure 1:**
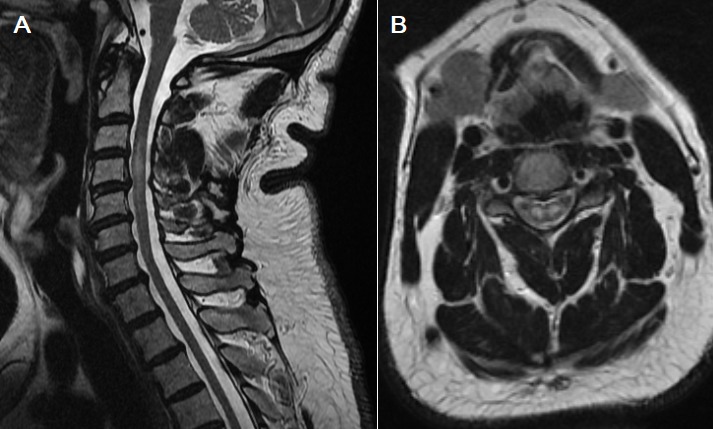
A) IRM médullaire en coupe sagittal séquence T2: hyper signal de la moelle cervical en regard de C3-C4; B) IRM médullaire en coupe axiale passant par C3 en pondération T2: hyper signal central des cornes antérieurs de la moelle en « yeux de serpents

